# Value-Added Pastry Cream Enriched with Microencapsulated Bioactive Compounds from Eggplant (*Solanum melongena* L.) Peel

**DOI:** 10.3390/antiox9040351

**Published:** 2020-04-23

**Authors:** Georgiana Horincar, Elena Enachi, Vasilica Barbu, Doina Georgeta Andronoiu, Gabriela Râpeanu, Nicoleta Stănciuc, Iuliana Aprodu

**Affiliations:** Faculty of Food Science and Engineering, Dunărea de Jos University of Galati, Domnească Street 111, 800201 Galati, Romania; georgiana.parfene@ugal.ro (G.H.); elena.ionita@ugal.ro (E.E.); vasilica.barbu@ugal.ro (V.B.); georgeta.andronoiu@ugal.ro (D.G.A.); gabriela.rapeanu@ugal.ro (G.R.); nicoleta.stanciuc@ugal.ro (N.S.)

**Keywords:** bioactive compounds, microencapsulation, antioxidant activity, eggplant, pastry cream

## Abstract

In this study, antioxidant-rich eggplant peel extract was used to obtain a value-added pastry cream. In order to reduce the susceptibility to degradation, microencapsulation of the biologically active compounds from the eggplant peel was first performed. The microencapsulated bioactive compounds powder (MBC) obtained through freeze-drying retained about 94.31% of the anthocyanins present in the extract, was rich in phenolic compounds, and displayed a high antioxidant activity. The purple colored powder was added to the pastry cream in different concentrations (5% and 10%), allowing significant increase of total phenolic content and antioxidant activity, which were rather stable over 72 h of storage under refrigeration conditions. Sensory evaluation indicated that addition of MBC resulted in improved color and overall acceptability of the pastry cream formulation. All pastry cream samples exhibited rheological behavior specific to the weak gel-like structures, with increasing values of storage modulus with MBC addition. The instrumental texture analysis showed that MBC addition to the pastry cream slightly decreased the firmness and improved the chewiness of the samples.

## 1. Introduction

Vegetal by-products from agro-food industry differ in their origin and include different components of plants, such as peel, stem, leaf, seed, kernel, etc. [1,2]. Vegetal by-products are rich sources of functional compounds such as fibers, minerals, vitamins, phytochemicals, and antioxidants, among others [3,4,5]. Functional ingredients can be obtained from vegetal by-products and can act as promising vehicles for the nutritional improvement of food and may also enhance their health promoting properties [6]. Pastry products are consumed by all people and might therefore have an important role in human nutrition. The addition of bioactive ingredients to pastry products has resulted in increased popularity among consumers due to the ability to reduce the risk of chronic diseases beyond nutritional functions [7]. 

Eggplant (*Solanum melongena* L.) is widely used for preparing various fresh, frozen, or canned food products, and it is ranked amongst the top 10 vegetables in terms of antioxidant activity due to the high level of phytochemicals such as anthocyanins, flavonoids, and phenolic compounds [8,9]. Eggplant peels, which are a vegetal by-product, possess higher content of total phenolic compounds compared to pulp and whole fruit [9]. Although eggplant peels are an important source of bioactive compounds, they are not usually used for recovering the natural pigments (anthocyanins) responsible for the purple color of eggplant and the phenolic compounds, which exhibit high antioxidant activity [9]. These bioactive compounds extracted from eggplant peel are an appealing alternative to chemical additives and can be used to improve the biological value of different food systems. Among anthocyanin pigments, nasunin (delphinidin-3-p-coumaroylrutinoside-5-glucoside) is the major compound found in the eggplants [10] and has the strongest in vitro antioxidant activity. 

Other anthocyanins found in the eggplant are delphininidin-3-rutinoside, delphininidin-3-glucoside, cyanidin-3-rutinoside, and petunidin-3-rutinoside [11,12,13]. The predominant phenolic compound found in all eggplant varieties is chlorogenic acid [14]. Phenolic compounds are known to display a wide range of biological activities, such as anti-inflammatory, antimicrobial, or anti-carcinogenic activities [15,16]. 

Anthocyanins are a group of natural pigments responsible for the red-blue color of many fruits and vegetables and can be used as natural food colorants [17]. However, the utilization of anthocyanins as food natural additives is limited because of their low stability. After extraction, these compounds are susceptible to degradation under certain conditions of pH, presence of metal ions, exposure to light and UV, temperature, oxygen, and enzymatic activity. Several studies have reported the wide range of biological activities of anthocyanins extracted from different vegetal sources and their susceptibility to environmental and chemical factors [8,14]. To reduce their susceptibility and achieve controlled release at the targeted site, the microencapsulation technique has been successfully applied [18]. When looking for stabilizing different matrices, microencapsulation can be regarded as appropriate means for overcoming these limitations. Thus, incorporation of anthocyanins in different coating materials allows their preservation while processing and storing the foods [18]. Moreover, encapsulated anthocyanins are great alternatives for synthetic dyes because of their attractive red-purple color and water solubility, which allows them to be incorporated into foods [19]. Several methods have been reported for microencapsulation of anthocyanins from different sources, including mechanical methods (spray drying and freeze-drying) and chemical methods (interfacial polymerization and coacervation) [18,20]. However, few studies have focused on the encapsulation of eggplant peel anthocyanins and incorporating them in food matrices [21].

The ideal coating materials used for bioactive compounds microencapsulation are biocompatible, non-toxic, and low cost [18]. Whey protein has already been used in many studies for the encapsulation of bioactive compounds [22,23]. Whey protein is regarded as a nutrient source providing essential amino acids and are versatile in solubility, gelation, film formation, foaming, emulsification, and water binding capacity [18]. Another coating material successfully used for microencapsulation of biologically active compounds is acacia gum, a polysaccharide that possess low viscosity at high solid content and in aqueous solution. Acacia gum has good emulsifying ability, is cheap, and is useful for enhancing the water solubility of hydrophobic compounds, which is vital for increasing their bioavailability [18].

Pastry creams of various colors, textures, and flavors are largely used in confectionery and are highly appreciated by consumers. Most assortments of pastry creams are obtained by using dyes, flavors, emulsifying agents, and other chemically synthesized food additives. This study aimed to obtain a pastry cream out of natural ingredients, without adding synthetic additives that might negatively impact consumer health. To the best of our knowledge, there are no available studies on obtaining value-added pastry creams using microencapsulated eggplant peel extract as a natural dye and source of bioactive compounds. The use of eggplant extract in the composition of pastry cream can contribute to increasing the nutritional value of the product and, therefore, the quality of life. Therefore, the objectives of this study were (1) to encapsulate bioactive compounds from eggplant peel through freeze-drying using whey protein isolate and acacia gum as a matrix, followed by characterization of the powders in terms of microencapsulation efficiency, phytochemical content, antioxidant activity, and morphological structure; (2) to obtain a value-added pastry cream by incorporation of the encapsulated eggplant peel extract. The obtained pastry cream samples were characterized in terms of phytochemical stability and antioxidant activity with their storage, texture, color, rheological, and sensory properties.

## 2. Materials and Methods 

### 2.1. Materials

Eggplants (*Solanum melongena* L.) were purchased from a local market (Galati, Romania) in September 2019. Whey protein isolate (WPI) (protein content of 95%) was purchased from Fonterra (Auckland, New Zealand). Acacia gum, 2,2-diphenyl-1-picrylhydrazyl (DPPH), 6-hydroxy-2,5,7,8-tetramethylchromane-2-carboxylic acid (Trolox), and Folin–Ciocalteu reagent were obtained from Sigma Aldrich (Steinheim, Germany). 

### 2.2. Phytochemical Extraction and Characterization of Eggplant Peel Extract 

The extraction of phytochemicals from freeze-dried eggplant peel was performed according to the procedure described in our previous study [13]. The resulting eggplant peel extract (EPE) was used for the microencapsulation experiment.

The phytochemical characterization of EPE was performed in terms of total monomeric anthocyanins (TMA), total phenolic content (TPC), total flavonoid content (TFC), and antioxidant activity, as described by Turturică et al. [24]. In order to quantify the TMA, TPC, and TFC, the extraction was performed by vigorously mixing, by means of a Vortex mixer, a suspension consisting of 10 mg of EPE and 5 mL ethanol 70%. The pH differential method was used to determinate the TMA content, expressed as milligrams of delphinidin-3-glucoside equivalents (D3GE) per gram of dry weight (DW) of extract (mg D3GE/g DW) [24,25]. The TFC was determined using a colorimetric method based on the aluminum chloride capacity of forming stable acid complexes with flavanols, and the results were expressed as milligrams of catechin equivalents per gram of dry weight of extract (mg CE/g DW) [24,26]. The Folin–Ciocalteu method was used to determine the TPC, expressed as milligrams gallic acid equivalents per gram of dry weight of extract (mg GAE/g DW), as describe by Turturică et al. [24]. The DPPH method was used to assess the antioxidant activity [27], and the results were expressed as millimoles of Trolox equivalents per gram of dry weight (mM TE/g DW).

### 2.3. Phytochemical Microencapsulation and Characterization of the Powder

The procedure previously designed by our group was used to microencapsulate the bioactive compounds from EPE in WPI and acacia gum [23]. The WPI (2.5%) and acacia gum (1%) were separately suspended in ultrapure water and stirred at 300 rpm with a mechanical stirrer for 4 h at room temperature (25 °C) to ensure complete hydration. The WPI and polymer solutions were mixed at a 2:1 ratio and further homogenized at 100 rpm for an additional 3 min. A volume of 30 mL of EPE solution (29.66 mg EPE/mL ultrapure water) was then added to 150 mL WPI and acacia gum solution, and the mixture was stirred at 100 rpm for 1 h at 40 °C. All homogenization steps were performed by using a mechanical stirrer with heating (IKA RCT Basic, Staufen, Germany). After appropriate mixing, the pH of the sample was adjusted to 4.5 with 1 N HCl until the mixture was a purple color. The Inolab 7110 (WTW, Weilheim, Germany) pH meter was used for pH measurements. The sample was then frozen at −70 °C, and the ice crystals were removed by freeze-drying (CHRIST Alpha 1–4 LD plus, Osterode am Harz, Germany) at −42 °C under a pressure of 0.10 mbar for 72 h. Afterwards, the microencapsulated bioactive compounds powder (MBC) was collected and kept at 5 °C until analysis.

For further phytochemical characterization, 50 mg of MBC was suspended in 5 mL of ultrapure water and was further subjected to sonication in an ultrasonic water bath (MRC Scientific Instruments) at 40 kHz and 40 °C for 10 min. The resulting solution was used for phytochemical characterization in terms of TMA, TPC, TFC, and antioxidant activity, as described by Turturică et al. [24].

### 2.4. Efficiency of Anthocyanin Encapsulation

In order to evaluate the encapsulation efficiency (EE) of anthocyanins, the method described by Akhavan Mahdavi et al. [28] was used, based on the difference between the total anthocyanin content (TAC) and the anthocyanin content located in the microcapsule surface (SAC). Briefly, in order to quantify the TAC, 50 mg of MBC was mixed with 5 mL of a mixture of ethanol, acetic acid, and water (50:8:42). The mixture was agitated using a Vortex for 1 min and then ultrasonicated for 20 min in order to destroy the microcapsules. After that, the mixture was centrifuged at 14,000 rpm for 10 min at 10°C and then filtered. In order to extract the SAC, 50 mg MBC was mixed with 5 mL ethanol: methanol (1:1) and was stirred at room temperature for 1 min, followed by centrifugation at 14,000 rpm for 10 min at 10 °C. After phase separation, the clear supernatant was collected and filtered through 0.25 μm Millipore membranes. The quantification of anthocyanins from MBC was performed by using the pH-differential method [29].

EE was calculated according to Equation (1) using the results from total (TAC) and surface (SAC) anthocyanin contents:

EE% = (TAC−SAC)/TAC × 100(1)

### 2.5. Preparation of Pastry Cream Supplemented with MBC 

Pastry cream was obtained from the following ingredients: milk with 1.5% fat (62.89%), sugar (11.02%), white wheat flour (12.24%), lemon juice (1.5%), butter with 65% fat (11.02%), and vanilla essence (0.06%). At first, flour, sugar, and milk were manually mixed by means of a spatula at 100 °C for 20 min in order to allow the gelatinization of flour starch, such as to get optimal creamy consistency. The mixture was cooled down, and the butter was gradually added to the mixture at room temperature. The MBC was further added to the pastry cream at two different concentrations of 5% and 10%, while continuously mixing, until obtaining the homogeneous purple samples coded PC-5 and PC-10, respectively. It should be noted that the complete and even distribution of MBC within the pastry cream is very challenging and might influence the homogeneity of the bioactive compounds within samples. Control cream was prepared in the same way, without adding the MBC. All cream samples were stored at 5 °C before the measurements. The TMA, TPC, TFC, and antioxidant activity of the pastry cream samples were determined using the methods presented in Section 2.2.

### 2.6. Confocal Laser Scanning Microscopy

The confocal analysis by means of a Zeiss Axio Observer Z1 (Carl Zeiss Microscopy GmbH, Köln, Germany) confocal inverted microscope (LSM 710 model) was used to observe the features of MBC. The microscope was equipped with the following laser scanning systems: diode laser (405 nm), Ar laser (458, 488, and 514 nm), DPSS (561 nm pumped solid-state diodes), and HeNe laser (633 nm). The distribution of the pigments into the complex matrix was observed using a 20× apochromatic objective and two types of magnification, 0.6 and 1. MBC was analyzed in its native state and also fluorescently labeled with two dyes, DAPI (1 µg/mL) and Red Congo (40 µM), in a ratio of 3:1:1. Furthermore, the comparative confocal microscopy analysis was also carried out with the experimental pastry creams in order to capture the structural, textural, and compositional changes. The pastry creams variants were observed in their native state and stained with the two fluorophores dyes, and the obtained 3D images were rendered and analyzed with the ZEN 2012 SP1 software (Black Edition, Carl Zeiss Microscopy GmbH, Jena, Germany).

### 2.7. Textural Profile Analysis

Texture Profile Analysis (TPA) performed with a CT3-1000 Texture Analyser (Brookfield Ametek, MA, USA) was used to evaluate the textural properties of the pastry cream samples. The method consisted of a double penetration into the sample of a 25.4 mm diameter acrylic cylinder, until 10 mm depth was reached. The testing speed was 1mm/s, the trigger load was 0.067 N, and the load cell was 9.8 N. TexturePro CT V1.5 software was used to register the force-deformation parameters and to calculate the textural parameters of firmness, adhesiveness, cohesiveness, springiness, and chewiness. Three determinations for each sample were done. The samples were packed into cylindrical plastic containers with 25 mm diameter and 45 mm height (the samples height was 32 mm) and kept at refrigeration temperatures (4–6 °C) until testing.

### 2.8. Rheological Properties

Rheological properties of the pastry cream samples were measured using an AR2000ex Rheometer (TA Instruments, New Castle, DE, USA) equipped with a Peltier temperature control system. All measurements were performed at 10 °C using a cone–plate geometry (40 mm in diameter, 2° cone angle) and a closing gap of 2000 μm.

The stepped flow test was employed to study the flow behavior of pastry cream samples. The shear rate was gradually increased from 0.1 to 100 s^−1^, and the obtained results were analyzed using TA Rheology Advantage Data Analysis Software V 4.8.3. (TA Instruments, New Castle, DE, USA).

The consistency coefficient and the flow behavior index values were calculated were determined by fitting the flow data to the Ostwald–de Waele model

η = K·(γ˙)^n−1^ (2)

where η is the apparent viscosity (Pa·s), K is the consistency index (Pa·s^n^), γ˙is the shear rate (s^−1^), and n is rate index, also known as flow behavior index (dimensionless).

When performing low-amplitude dynamic shearing tests, the storage modulus (G′) and loss modulus (G″) were registered. The strain sweep tests were performed at an oscillatory frequency of 1 Hz while increasing strain from 0.01% to 100%. This test allowed identifying the linear viscoelastic region (LVR) for all investigated samples. Further dynamic frequency sweeps were performed at a constant strain within the LVR by increasing the frequency in the 0.1–100 Hz domain.

### 2.9. Color Measurements

Color attributes of the pastry cream samples with different percentages of MBC were measured using the CR300 Chroma Meter (Konica Minolta, Munich, Germany) equipped with a D65 Illuminant. The measurements of the CIELAB color parameters (L*, a*, and b*) were taken in triplicate after calibrating the instrument against a white plate.

### 2.10. Sensory Analysis

The sensory evaluation of pastry cream samples was done with 15 untrained consumers with ages ranging from 22 to 60 years old (90% women and 10% men). An orientation session was provided to the panelists prior to sensory analysis. The 9-point Hedonic assessment was used with scores from 1 (very dislike) to 9 (very like), as described by Aggarwal et al. [30]. Consumers evaluated the appearance, color, consistency, taste, aroma, and smell of the cream samples with MBC and control sample (without MBC), respectively. The samples were served at room temperature.

### 2.11. Statistical Analysis

The experiment was run in duplicate, and all measurements were carried out at least in triplicate. The results are presented as mean ± standard deviation. Analysis of variance (ANOVA) was employed to identify significant differences among results. Statistical relationships were established based on Pearson’s correlation coefficients. One-way ANOVA and Tukey’s test with a 95% confidence interval were applied using Minitab 18 software; *p* < 0.05 was considered to be statistically significant.

## 3. Results and Discussion

### 3.1. Phytochemical Extraction and Characterization of EPE

The eggplant peel extract was characterized in terms of phytochemical content and antioxidant activity. The TMA, TFC, and TPC were 0.55 ± 0.01 mg D3GE/g DW, 5.20 ± 0.91 mg CE/g DW, and 13.65 ± 0.31 mg GAE/g DW, respectively. The extract showed an inhibition of 46.34 ± 0.17%, corresponding to a DPPH radical scavenging capacity of 183.14 ± 2.31 mM TE/g DW. These results are in agreement with Condurache et al. [22], who characterized the EPE obtained by ultrasound-assisted extraction and reported TMA content of 0.58 ± 0.03 mg D3G/g DW, TFC of 4.48 ± 0.80 mg CE/g DW, and TPC of 13.64 ± 0.19 mg GAE/g DW. Other authors investigated the phytochemical characteristics of EPE and reported variable TMA, TFC, and TPC, which are due to the difference regarding the cultivar, environment, and extraction conditions. For example, Dranca and Oroian [31] reported the TMA for different eggplant genotypes ranging from 80 to 850 mg/kg peel, with variability due to agronomic and genetic factors, intensity, and type of light and temperature. Jung et al. [32] reported TFC of 6.19 ± 0.28 mg CE/g eggplant peel extract, and Ferarsa et al. [33] reported the TPC of 29.011 mg GAE/g DM for the eggplant peel extract using ultrasound-assisted extraction.

### 3.2. Microencapsulation Efficiency and Characterization of the MBC

The experimental results indicated that the microencapsulation efficiency of anthocyanins was 94.31 ± 3.32%. Our results are in agreement with data from other studies that reported values for encapsulation efficiency of 91–97% when encapsulating anthocyanins in maltodextrin and Arabic gum [34], or 94–99% for grape skin anthocyanins microencapsulated in WPI, acacia gum, and pectin [23]. In the present study, the microencapsulated powder showed TMA of 0.837 ± 0.086 mg D3GE/g DW, TFC of 0.277 ± 0.081mg CE/g DW, TPC of 5.863 ± 0.936 mg GAE/g DW, and antioxidant activity of 45.522 ± 6.789 mM TE/g DW. The higher content of TMA found in the MBC compared to the EPA might be explained by the differences in terms of the solvent used for the extraction of the biologically active compounds. In the case of EPE, the extraction was performed while vigorously mixing the samples suspended in 70% ethanol, whereas in the case of MBC, the ultrasound-assisted extraction in ultrapure water was employed, which is known to be efficient in destroying the microcapsules and releasing of encapsulated compounds. Glycosylated anthocyanins are highly soluble in water, and solubility decreases with increasing degrees of acylation [35]. On the other hand, TFC and TPC have better solubility in 70% ethanol than in ultrapure water.

Our results regarding the phytochemical contents of MBC are comparable with those reported by Condurache et al. [22], who used different combinations of carboxymethylcellulose (CMC), pectin (P), WPI, and whey protein hydrolysate (WPH) for the encapsulation of bioactive compounds from EPE. They reported TMA contents ranging between 0.52 and 0.89 mg D3G/g DW, TFC in the range of 20.81–8.67 mg CE/g DW, and TPC within the range 62.49–69.15 mg GAE/g DW. The TMA content of MBC fell within the range reported by Condurache et al. [22], suggesting that the mixture of WPI and acacia gum is as efficient as the combinations of CMC, P, WPI, and WPH for encapsulating the anthocyanins from EPE. On the other hand, the TFC and TPC of MBC were lower compared to those reported by Condurache et al. [22]. It appears that the encapsulation matrix containing CMC, P, WPI, and WPH was more efficient in forming colloidal particles, which successfully encapsulated the flavonoids and other phenolic compounds, compared to the matrix consisting of WPI and acacia gum. Instead, our results in terms of TPC are similar with those reported by Sarabanti et al. [21] who found that the TPC of encapsulated eggplant extract using maltodextrin and Arabic gum (1:1) ranged between 4.57 and 5.22 mg GAE/g powder. Finally, the antioxidant activity of MBC was slightly lower compared with the data reported by Condurache et al. [22] (antioxidant activity of 55.43 ± 7.17 to 64.58 ± 0.49 mM TE/g DW).

### 3.3. Pastry Cream Enriched with MBC

The pastry cream samples enriched with different amounts of MBC were stored for 72 h at 5 ± 0.5 °C, and the phytochemical composition and antioxidant activity were evaluated every 24 h (Table 1). As expected, the content of bioactive compounds increased with the amount of MBC added to the pastry cream. The TPC and TFC of the control sample (no added MBC) decreased significantly (*p* < 0.05) over the tested storage period (Table 1). The addition of microencapsulated eggplant peel extract to the cream composition resulted in a significant increase (*p* < 0.05) of the bioactive compounds (TMA and TFC) levels, which were rather stable over 72 h of storage under refrigeration conditions. A significant decrease of the TFC was obtained for the control pastry over 72 h of storage, whereas in the case of samples supplemented with different levels of MBC, no significant variation of TFC was observed (Table 1). These results suggest that the MBC powder is able to interact with the flavonoids originating from the ingredients used for preparing the control pastry, improving their stability. Only a slight decrease of TMA level of pastry cream samples was noticed when extending the storage period up to 72 h (Table 1). Regarding the TPC values of the control sample, a significant decrease was noticed over the entire storage period (Table 1). An improved TPC stability was registered for the pastry cream samples supplemented with different amounts of MBC; only a slight decrease was noticed after 24 h of storage. Moreover, at the end of the storage period, the samples PC-5 and PC-10 had significantly higher levels of bioactive compounds (*p* < 0.05), compared to the control sample (Table 1). In addition, the antioxidant activity of the pastry cream samples supplemented with MBC was higher compared to the control because of the higher levels of bioactive compounds coming from the EPE. Only a slight decrease of the antioxidant activities of the PC-5 and PC-10 samples was observed after 24 h of storage (Table 1).

The incorporation of MBC into pastry cream led to significant enrichment in TPC, TFC, and TMA. The presence of flavonoids and other phenolic compounds in the control sample was due to the contribution of the main ingredients used to prepare the pastry cream, which lack anthocyanins. Therefore, the TMA content of the PC-5 and PC-10 samples arose from the MBC (Table 1). The bioactive compounds extracted from eggplant peel positively influenced the in vitro antioxidant activity of the enriched pastry cream. They showed an antioxidant activity significantly higher than that of the control pastry cream.

The results of the present study are in agreement with our previous findings on the microencapsulated anthocyanin-rich extract derived from black rice used to enrich pastry cream [36]. Aprodu et al. [36] showed that pastry cream samples enriched with microencapsulated black rice extract had, over the entire tested storage period of 48 h, higher contents of bioactive compounds (TAC 0.08–0.12 mg C3G/g DW; TFC 0.45–1.37mg CE/g DW; TPC 1.00–2.01 mg GAE/g DW) and a significant antioxidant activity (40.55–42.30 M TE/g DW) compared to the control, which demonstrates the added value.

### 3.4. Morphological Structure of the MBC and Pastry Creams

**Figure 1:**
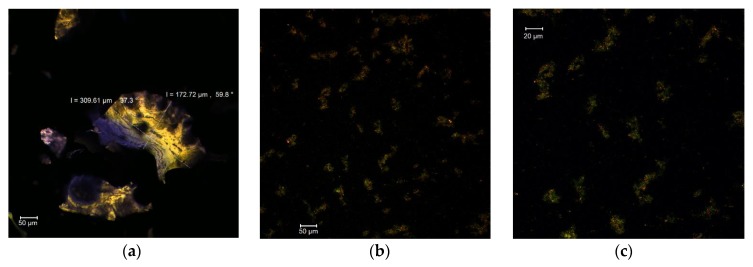
Confocal laser scanning microscopy images of MBC: native state MBC (**a**); fluorescently labelled MBC, magnification 0.6—707.3 µm × 707.3 µm, with pixel size of 0.60 µm (**b**); and fluorescently labelled MBC, magnification 1—424.4 µm × 424.4 µm with pixel size of 0.36 µm (**c**).

The confocal analysis of MBC in the native form revealed the presence of several fine formations with irregular edges and rather unitary appearance, having dimensions in the 172–309 µm range and autofluorescence in the green-yellow domain (Figure 1a). Regarding the encapsulating matrix, consisting of whey protein isolate and acacia gum, it displayed an emission in the blue domain. The fluorescently labeled samples revealed the presence of the anthocyanins from MBC as small microparticles (size 1–3 µm), with an emission around the wavelength of 585 nm within the biopolymer matrix (Figure 1b,c).

**Figure 2:**
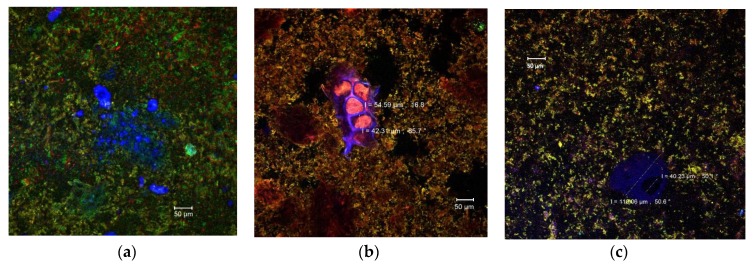
Confocal laser scanning microscopy images of the pastry cream samples: Control (**a**), PC-5 (**b**), and PC-10 (**c**).

Due to the diversity of the ingredients used in the pastry cream recipe (milk, sugar, butter, wheat flour, etc.) that interact with each other, it is extremely difficult to identify each component separately by means of confocal laser scanning microscopy. Singh and Mishra [37] showed that monomeric anthocyanins display emissions in a wide range of wavelengths (550–650 nm), but the maximum emission was recorded at 585 nm (yellow to orange). Compared to the control pastry cream (Figure 2a), numerous yellow-orange microparticles could be observed in the samples with added MBC. These microparticles were aggregated into clusters of different sizes and dispersed more or less uniformly in the complex matrix of the product (Figure 2b,c). The microparticles appeared to be larger in the 10% MBC sample (Figure 2c). Also, the anthocyanins rich microencapsulated powder which contains bioactive compounds extracted from the eggplant peels also came with fibers from the fragments of plant tissue, which were frequently found in the product (Figure 2b). Thus, parenchymal cells with diameters between 42.31 and 54.59 μm can be observed, with intact cellulose walls (in blue) and an EPE anthocyanins rich cellular content (Figure 2b). In Figure 2c some large glycoprotein conglomerates (in blue), up to at 110.06 µm, were also present most likely from the pastry cream ingredients, milk, or flour.

### 3.5. Textural Properties of Pastry Cream

The results of the instrumental texture analysis are presented in Table 2. The addition of microencapsulated powder to the pastry cream determined a soft decrease of firmness by 1.22 N for PC-5 sample and 1.20 N for PC-10 sample, compared to the control sample. The firmness indicates the force necessary to induce a given deformation of the pastry cream samples [38]. The decrease of the firmness values with the level of MBC added to the pastry cream might be the result of the MBC interference with the gel network formation. The size of the MBC is higher compared to the protein molecules involved, together with the gelatinized starch granules, in defining the three-dimensional network of the heat-set gel. Therefore, MBC addition might result in disruption of the network.

On the other hand, addition of MBC to the pastry cream formulations had no significant impact on the other textural parameters such as adhesiveness, cohesiveness, springiness, and chewiness (Table 2). Adhesiveness is a measure of the work required to pull the compressive probe away from the sample [38]. Cohesiveness is an expression of the strength of the internal bonds making up the body of the product [38]. Addition of MBC caused no important changes in the cohesiveness values of the pastry cream. Springiness is a measure of sample elasticity, and according to the results presented in Table 2, PC-10 sample presented the highest elasticity (8.39 mm). The control sample and PC-10 sample has similar values of chewiness, which is the energy required to disintegrate the sample during mastication [38].

### 3.6. Rheological Characterization of MBC Enriched Creams

**Figure 3:**
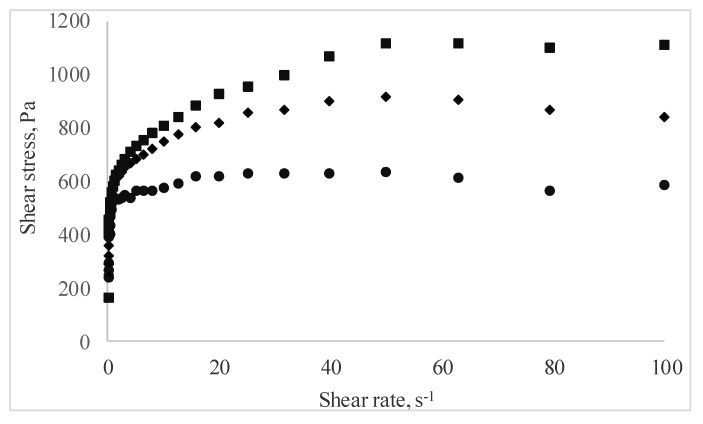
Curves of the pastry cream samples supplemented with different amounts of MBC (Control—full circles, 5% MBC—full diamonds, 10% MBC—full square).

The rheological behavior of the pastry cream samples under flow conditions is presented in Figure 3 as shear stress vs. shear rate data. Regardless of MBC addition, all pastry cream samples displayed shear thinning behavior. The shear rate increased, and the apparent viscosity of the samples decreased over the entire tested shear rate. This behavior is the result of the changes occurring at increasing shear rates in the structure of the macromolecular compounds agglomerated in the samples, and it is in agreement with the results reported by Toker et al. [39] who investigated the effect of addition and interaction between different gums on the rheological properties of dairy desserts.

The Ostwald–de Waele model was used to evaluate the rheological behavior, and the results indicated that all tested samples exhibited pseudoplastic behavior, with *n* values lower than 1. The consistency index, rate index, and the apparent viscosity increased with the solid fraction from MBC addition to the pastry cream sample (Table 3).

**Figure 4:**
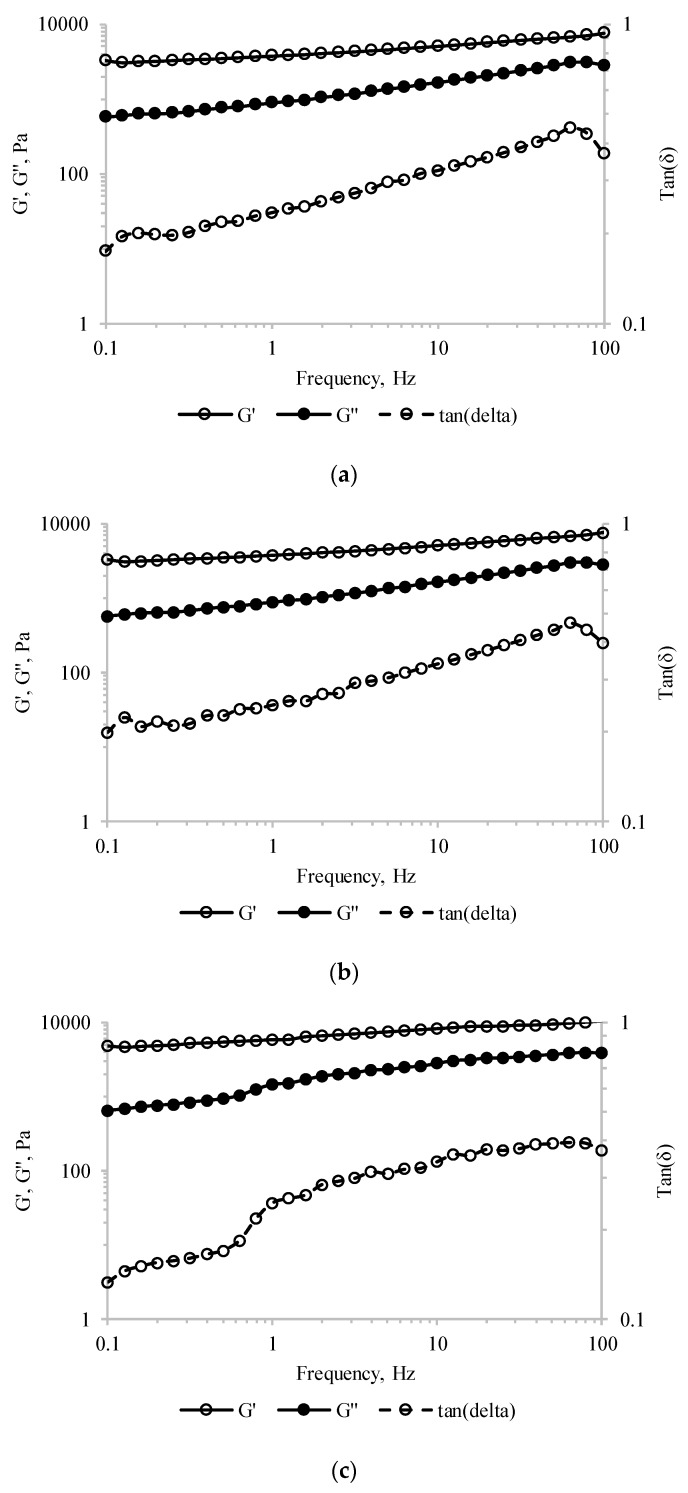
Storage modulus (*G*′), loss modulus (*G*″), and tan(δ) values of the pastry cream sample supplemented with different amounts of MBC: (**a**) Control—0% MBC, (**b**) 5% MBC, (**c**) 10% MBC.

Frequency sweep tests performed in the linear visco-elastic region were further used to asses information on the structural assembly of the tested material. The storage modulus (*G*′) values were higher than the loss modulus (G″) values, and both moduli increased over the entire frequency domain for all tested samples (Figure 4). These results indicated that, regardless of MBC addition, the pastry cream samples exhibited behavior specific to the weak gel-like structures. Similar observations were reported by Toker et al. [39] for the dairy desserts including 0.2% gums like carrageenan, alginate, guar and/or xanthan gums. Moreover, sub-unitary tan(*δ*) values were measured over the whole frequency range (Figure 4), suggesting that all pastry cream samples had elastic properties.

For the sake of comparison, the viscoelastic parameters (*G*′, *G*″, complex modulus—G*, and tan *δ*) at 1 Hz frequency are presented in Table 3. The increasing values of *G*′ with MBC addition suggest stronger particle–particle interactions involved in stabilizing the network-type structure [39]. Moreover, the increase of the complex modulus (G*^2^ = G′^2^ + G″^2^) of PC-10 by ~54%, compared to the control samples, is a measure of the enhanced resistance to deformation of the material [39].

### 3.7. Color Parameters of MBC and Enriched Creams

The purple color of the eggplants characterized the presence of important amounts of anthocyanins, mainly to delphinidin-based pigments, whereas the darkening effect appeared as the result of the presence of chlorophylls [40]. The chromatic data registered for the MBC in the CIELAB color space indicated the red color (a* of 14.64) and the presence of blue hue (b* of −0.33), suggesting the presence important amounts of anthocyanins in the sample. The MBC addition to the pastry cream resulted in important color changes (Table 4).

The lightness of the samples decreased significantly (*p* < 0.05) with the amount of MBC added to the pastry cream (Table 4). Because of the lack of anthocyanins, a greenish shade (a* of −2.71) was identified with the yellow color (b* of 24.34), which prevailed in the case of the control sample. As a result of increasing the amount of MBC added to the pastry cream sample, which provided important amounts of eggplant peel pigments, the a* values shifted from green to red domain (a* of 14.25 for PC-10), whereas the yellow color faded and b* reached 8.82 (Table 4).

### 3.8. Sensorial Analysis of MBC Enriched Creams

**Figure 5:**
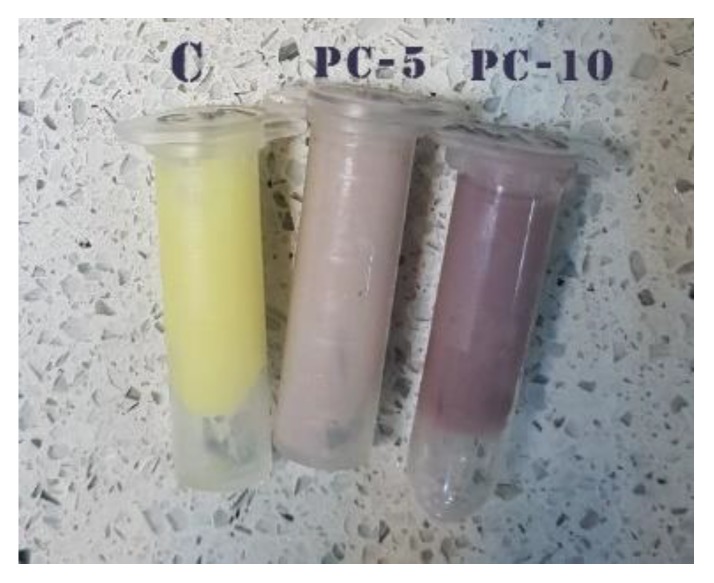
Pastry cream samples enriched with different levels of MBC powder (Control—0% MBC; PC-5—5% MBC; PC-10—10% MBC).

The sensory attributes registered for pastry cream samples enriched with different levels of MBC are presented in Table 5. Among the sensory indicators, color is the first indicator reflecting the quality of a product that attracts a consumer’s attention [41]. The color of pastry cream was significantly influenced by the concentration of MBC (*p* < 0.05) (Table 4). Thus, the PC-10 sample with 10% MBC got the highest score in terms of color, being followed by PC-5 with 5% MBC (Table 5). The enrichment of pastry cream with MBC led to creating an attractive purple color in the product as a result of increasing amounts of anthocyanins (Figure 5).

On the other hand, the concentrations of MBC added to the cream samples did not have any effect on flavor and smell, but the taste of the product was affected by the high level of powder (10%). Also, it can be observed that addition of higher contents of MBC to the pastry cream resulted in slightly increased consistency and appearance scores. No significant differences among the three samples in terms of overall acceptance were registered (Table 5). In addition, the PC-10 sample with 10% MBC was more appreciated by the panelists due to the color (Figure 5).

The results are in agreement with Sarabandi et al. [21], who evaluated the sensory properties of fortified candy enriched with microencapsulated eggplant peel extract and indicated that addition of 1.5% powder improved the color properties and overall acceptability of the product. Moreover, Sarabanti et al. [21] reported that microencapsulated eggplant peel extract could be used as a natural ingredient in production of functional foods, confectionery, sauces, chocolates, jelly, ice cream, candy products, and a variety of instant drinks and powders to enhance their nutraceutical value.

## 4. Conclusions

Eggplant peel is a by-product obtained during processing of eggplant products and is an important source of bioactive compounds. In this study, the bioactive compounds from eggplant peel were microencapsulated by freeze-drying using a mixture of whey proteins and acacia gum as wall material. The obtained MBC was rich in phenolic compounds and had high antioxidant activity. The functionality of the MBC was further tested by addition to a pastry cream formulation. The antioxidant activity of the pastry cream samples increased with the level of MBC, and the encapsulation matrix ensured the high stability of the biologically active compounds in the food matrix during three days of storage. The pastry cream enriched with MBC had a higher nutritional quality than the control sample due to the increase of polyphenol content. MBC addition caused a significant increase of redness value and decrease of yellowness of the pastry cream sample, as a result of the presence of important amounts of pigments extracted from eggplant peels. Sensory evaluation of value-added pastry cream indicated that addition of MBC powder improved color properties of the product. All pastry creams exhibited rheological behavior specific to a gel structure, with storage modulus values prevailing over the loss modulus values. Moreover, MBC addition to the pastry creams exhibited no significant influence on the adhesiveness, cohesiveness, springiness, and chewiness of the samples. It can be concluded that MBC can be used as a natural ingredient in the production of different value-added foods such as pastry, chocolate, jelly, ice cream and candy products.

## Figures and Tables

**Table 1:** Phytochemical characterization and antioxidant activity of pastry cream enriched with MBC (Control—0% MBC; PC-5—5% MBC; PC-10—10% MBC), during storage (72 h).

Sample	Storage Time (hours)	Bioactive Compounds			Antioxidant Activity
		TMA (mg D3G/g DW)	TFC (mg CE/g DW)	TPC (mg GAE/g DW)	DPPH (mM/g DW)
Control	0	Nd ^1^	0.334 ± 0.005 ^a^	0.633 ± 0.027 ^a^	0.099 ± 0.06 ^a^
	24	nd	0.282 ± 0.000 ^b^	0.475 ± 0.068 ^b^	0.088 ± 0.013 ^a^
	48	nd	0.162 ± 0.014 ^c^	0.373 ± 0.076 ^b^	0.073 ± 0.011 ^a^
	72	nd	0.141 ± 0.018 ^c^	0.215 ± 0.036 ^c^	0.065 ± 0.028 ^a^
PC-5	0	0.125 ± 0.011 ^a,2^	0.462 ± 0.009 ^a^	3.921 ± 0.131 ^a^	2.282 ± 0.030 ^a^
	24	0.115 ± 0.007 ^a,b^	0.462 ± 0.012 ^a^	3.887 ± 0.076 ^a^	1.809 ± 0.043 ^b^
	48	0.105 ± 0.009 ^a,b^	0.460 ± 0.020 ^a^	3.870 ± 0.064 ^a^	1.777 ± 0.007 ^b^
	72	0.100 ± 0.008 ^b^	0.455 ± 0.015 ^a^	3.794 ± 0.015 ^a^	1.765 ± 0.007 ^b^
PC-10	0	0.255 ± 0.017 ^a^	0.805 ± 0.019 ^a^	6.857 ± 0.132 ^a^	4.814 ± 0.007 ^a^
	24	0.247 ± 0.007 ^a^	0.807 ± 0.019 ^a^	6.769 ± 0.081 ^a,b^	4.419 ± 0.025 ^b^
	48	0.226 ± 0.014 ^a^	0.801 ± 0.003 ^a^	6.593 ± 0.147 ^a,b^	4.407 ± 0.022 ^b^
	72	0.222 ± 0.012 ^a^	0.780 ± 0.020 ^a^	6.531 ± 0.100 ^b^	4.401 ± 0.034 ^b^

^1^ nd—not detected, ^2^ Within a set of determinations, means of the same sample that do not share a superscript letter (a, b or c) are significantly different at *p* < 0.05.

**Table 2:** Results of instrumental texture analysis of the pastry cream samples enriched with different levels of MBC (Control—0% MBC; PC-5—5% MBC; PC-10—10% MBC).

Textural Parameter	Control	PC-5	PC-10
Firmness, N	7.45 ± 0.25 ^a,1^	6.23 ± 0.26 ^b^	6.25 ± 0.34 ^b^
Adhesiveness, mJ	40.93 ± 2.33 ^a^	32.67 ± 6.17 ^a^	36.35 ± 6.80 ^a^
Cohesiveness	0.59 ± 0.03 ^a^	0.57 ± 0.10 ^a^	0.62 ± 0.03 ^a^
Springiness, mm	7.86 ± 0.35 ^a^	7.67 ± 1.12 ^a^	8.39 ± 0.56 ^a^
Chewiness, mJ	28.93 ± 4.18 ^a^	28.67 ± 9.29 ^a^	30.06 ± 2.15 ^a^

^1^ Means within a line that do not share a superscript letter (a or b) are significantly different at *p* < 0.05.

**Table 3:** The effect of microencapsulated bioactive compounds powder addition on the flow behavior parameters (consistency index—*K*, flow behavior index—n and apparent viscosity at shear rate of 50 s^−1^—*Ƞ_50_*) and viscoelastic parameters (storage modulus—*G*′, loss modulus—*G″*, complex modulus—G*, and tan *δ,* at frequency of 1 Hz) and of pastry cream samples (Control—0% MBC, PC-5—5% MBC, PC-10—10% MBC).

Rheological Parameter	Control	PC-5	PC-10
Flow behavior parameters			
*K*, Pa·s^n^	429.69	501.89	537.63
*n*	0.002	0.161	0.182
*R* ^2^	0.962	0.996	0.992
*Ƞ_50_*, Pa·s	8.67	18.23	22.26
Viscoelastic parameters			
*G′*, Pa	3370	3767	5191
*G″*, Pa	830.2	882	1204
G*, Pa	3471	3869	5329
tan *δ*	0.2464	0.2341	0.232

*R*^2^—correlation coefficient.

**Table 4:** The CIELAB color parameters (L*—lightness; a*—green-to-red; b*—blue-to-yellow) of investigated MBC and pastry cream samples with different levels of MBC (Control—0% MBC; PC-5—5% MBC; PC-10—10% MBC).

Samples	Color Parameters		
	L*	a*	b*
MBC	62.69 ± 0.04	14.64 ± 0.06	−0.33 ± 0.02
Control	80.11 ± 0.08 ^a1^	−2.71 ± 0.09 ^c^	24.34 ± 0.01 ^a^
PC-5	64.74 ± 0.15 ^b^	12.05 ± 0.03 ^b^	13.04 ± 0.10 ^b^
PC-10	58.21 ± 0.08 ^c^	14.25 ± 0.12 ^a^	8.82 ± 0.15 ^c^

^1^ Means within a column that do not share a superscript letter (a, b or c) are significantly different at *p* < 0.05.

**Table 5:** Sensory scores (9-point Hedonic Scale) for the pastry cream enriched with MBC (Control—0% MBC; PC-5—5% MBC; PC-10—10% MBC).

Sample	Sensory Attributes						
	Appearance	Color	Consistency	Taste	Flavor	Smell	Overall Acceptability
Control	8.60 ± 0.63 ^a,1^	7.33 ± 0.98 ^c^	8.47 ± 0.74 ^a^	8.80 ± 0.41 ^a^	8.87 ± 0.35^a^	8.87 ± 0.35 ^a^	8.07 ± 0.88 ^a^
PC-5	8.67 ± 0.49 ^a^	8.33 ± 0.72 ^b^	8.53 ± 0.64 ^a^	8.60 ± 0.63 ^a^	8.87 ± 0.35^a^	8.80 ± 0.41 ^a^	8.53 ± 0.52 ^a^
PC-10	8.73 ± 0.46 ^a^	9.00 ± 0.00 ^a^	8.60 ± 0.63 ^a^	8.53 ± 0.64 ^a^	8.87 ± 0.35^a^	8.80 ± 0.56 ^a^	8.47 ± 0.74 ^a^

^1^ Means within a column that do not share a superscript letter (a, b or c) are significantly different at *p* < 0.05.
